# Direct Activation of Cystic Fibrosis Transmembrane Conductance Regulator Channels by 8-Cyclopentyl-1,3-dipropylxanthine (CPX) and 1,3-Diallyl-8-cyclohexylxanthine (DAX)

**DOI:** 10.1074/jbc.273.10.5727

**Published:** 1998-03-06

**Authors:** Nelson Arispe, Jianjie Ma, Kenneth A. Jacobson, Harvey B. Pollard

**Affiliations:** ‡ Institute for Molecular Medicine and Department of Anatomy and Cell Biology, Uniformed Services University School of Medicine (USUHS), Bethesda, Maryland 20814,; § Department of Physiology and Biophysics, Case-Western Reserve University, Cleveland, Ohio 44106,; ¶ Section on Bioorganic Chemistry, Laboratory of Chemistry, NIDDK, National Institutes of Health, Bethesda, Maryland 20892

## Abstract

8-Cyclopentyl-1,3-dipropylxanthine (CPX) and 1,3-diallyl-8-cyclohexylxanthine (DAX) are xanthine adenosine antagonists which activate chloride efflux from cells expressing either wild-type or mutant (ΔF508) cystic fibrosis transmembrane conductance regulator (CFTR). These drugs are active in extremely low concentrations, suggesting their possible therapeutic uses in treating cystic fibrosis. However, knowledge of the mechanism of action of these compounds is lacking. We report here that the same low concentrations of both CPX and DAX which activate chloride currents from cells also generate a profound activation of CFTR channels incorporated into planar lipid bilayers. The process of activation involves a pronounced increase in the total conductive time of the incorporated CFTR channels. The mechanism involves an increase in the frequency and duration of channel opening events. Thus, activation by these drugs of chloride efflux in cells very likely involves direct interaction of the drugs with the CFTR protein. We anticipate that this new information will contribute fundamentally to the rational development of these and related compounds for cystic fibrosis therapy.

Cystic fibrosis (CF)^1^ is the most common, fatal, autosomal recessive disease in the United States, affecting nearly one birth in 2000 (1, 2). The responsible gene has been identified as CFTR (cystic fibrosis transmembrane conductance regulator), and the vast majority of CF patients carry a deletion of phenylalanine from position 508 (ΔF508; Refs. 3–5). The physiological function of CFTR includes cAMP-activated chloride channel activity (6–9), and the mutation compromises the ability of CFTR to traffic out of the endoplasmic reticulum to its appropriate location on the apical plasma membrane (10). Repair of the mutation can be effected by transferring wild-type CFTR into the mutant cell (11), and the long term strategy for treatment of cystic fibrosis has thus become focused on gene therapy at the level of the whole organism. However, it has also been observed that the ΔF508 CFTR does have intrinsic channel activity (12, 13). Furthermore, incubation of some mutant cells at low temperature (14), or in chemical chaperones such as 1 m glycerol (15), do permit the mutant CFTR to traffic out of the endoplasmic reticulum to the plasma membrane. Once at the membrane, the mutant CFTR exhibits cAMP-activated chloride channel activity, thereby permitting functional repair. These data have thus been interpreted to indicate that the ΔF508-CFTR is intrinsically active, and more recent data have shown that in some cells a small but measurable portion of the mutant CFTR actually spontaneously traffics to the vicinity of the plasma membrane (16, 17). These data therefore suggest that an alternative way to overcome the reduced chloride transport in CF cells might be to find compounds which further activate the small portion of mutant CFTR chloride channels which have trafficked to the cell membrane.

The adenosine A_1_-receptor antagonist CPX (8-cyclopentyl-1,3- dipropylxanthine) has been shown to stimulate ^36^[Cl^−^] efflux from pancreatic CFPAC-1 cells (18) in the concentration range of 20–100 nm. These cells are homozygous for the ΔF508 genotype common to most cases of cystic fibrosis. Similar results were obtained with the CF tracheal epithelial cell line IB3–1 expressing the ΔF508 allele, and with recombinant mouse fibroblast NIH 3T3 cells (19). Schweibert *et al*. (20) have also shown that CPX (50 nm) activates outward chloride currents in whole cell patch studies of primary explants of nasal epithelial cells from homozygous ΔF508 CF patients as well as wild-type CFTR control cells. More recently, Haws *et al*. (17) showed that CPX could activate iodide efflux from recombinant cells expressing ΔF508 CFTR. Thus wherever the CFTR mutant has been expressed, or in some favorable cases the wild-type CFTR, an effect of CPX on chloride efflux can be demonstrated.

In considering how CPX activates wild-type CFTR or repairs mutant CFTR, it is possible that CPX action might either be directly on the CFTR molecule, or be secondary to binding of CPX to another protein (21). As indicated above, CPX is best known as an adenosine A_1_-receptor antagonist (22). However, Northern blot analysis and structure-activity relationship (“SAR”) studies with 26 different compounds indicate that the A_1_-receptor is not responsible for CPX action in CFPAC cells (23). As one useful example, 1,3-diallyl-8-cyclohexylxanthine (DAX), a poor A_1_ antagonist, is also highly potent and efficacious in stimulating chloride efflux from CFPAC-1 cells (23). Thus the site of action of these xanthines in stimulating chloride efflux appears to represent a novel site of action, possibly involving direct interaction with the CFTR molecule. Additional information consistent with this latter possibility are data indicating that radiolabeled CPX binds with high affinity to the recombinant first nucleotide-binding fold (NBF-1) of CFTR (24) and to a subdomain within NBF-1 (25).

To test this hypothesis directly at the single channel level, we have incorporated recombinant wild-type CFTR channels from HEK293 cell microsomal membranes into planar lipid bilayers, and tested whether CPX could activate chloride currents through CFTR channels. In addition, we tested whether DAX, another active CPX analogue, could also activate chloride currents through CFTR channels. We report here that both CPX and DAX potently activate cAMP-dependent CFTR chloride channels. Furthermore, both compounds affect CFTR channel kinetics differently, and in a manner predictable from their respective actions on chloride efflux from different cell types. Furthermore both CPX and DAX also bind with high affinity to the recombinant first nucleotide-binding fold domain (NBF-1) of CFTR (24). These data together indicate that the mechanism of CFTR channel activation very likely involves direct interaction of the drugs with the CFTR protein. We anticipate that this new information will contribute fundamentally to the rational development of these and related compounds for cystic fibrosis therapy.

## MATERIALS AND METHODS

### Expression of CFTR in HEK293 Cells—

Wild-type CFTR cDNA was subcloned into the eukaryotic expression vector pCEP4 (Invitrogen) between the *Nhe*I and *Xho*I restriction sites to create the recombinant vector, pCEP4(CFTR) (11). A human embryonic kidney cell line (HEK293-EBNA: Invitrogen) was used for the transfection and expression of CFTR protein (26–28). This cell line contains the vector pCMV-EBNA which constitutively expresses the Epstein-Barr virus EBNA-1 gene product which increases the transfection efficiency of Epstein-Barr virus-based vectors. The parent cell line was maintained in Dulbecco’s modified Eagle’s medium containing 10% fetal bovine serum and 1% glutamine. Geneticin (G418, 250 *μ*g/ml) was added to the cell culture media to maintain selection of the cells containing pCMV-EBNA vector until after CFTR gene transfer. pCEP4(CFTR) was then introduced to the cell using Lipofectin reagent (Life Technologies), and 2 days after transfection, the cells were passaged and selected for hygromycin resistance (hygromycin B, 260 *μ*g/ml). Three weeks after transfection, microsomal vesicles were isolated from transfected cells. The expression of CFTR protein was conformed by Western blot using an antibody against the R domain of CFTR (mAb 13–1, Genzyme; Ref. 28).

### Isolation of Microsomal Membranes from Cultured Cells—

Microsomal vesicles were isolated from HEK 293 cells expressing wild-type CFTR protein using a modified protocol of Gunderson and Kopito (29), as described previously (26, 30). Briefly, 12 × 75-cm^2^ flasks of HEK 293 cells transfected with pCEP4(CFTR) vectors were harvested. The cell pellet was resuspended in ice-cold hypotonic lysis buffer (10 mm HEPES/NaOH, pH 7.2, 1 mm EDTA, 5 *μ*m diisopropyl fluorophosphate, 10 *μ*g/ml pepstatin A, 10 *μ*g/ml aprotinin, and 10 mg/ml benzamidine) before lysis by 10 strokes in a tight-fitting Dounce glass homogenizer, followed by 15 strokes after the addition of an equal volume of sucrose buffer (500 mm sucrose, 10 mm HEPES/NaOH, pH 7.2). Microsomes were collected by centrifugation of a postnuclear supernatant (600 × *g* for 15 min) at 100,000 × *g* for 45 min, and resuspended in 1 ml of prephosphorylation buffer (250 mm sucrose, 10 mm HEPES/NaOH, pH 7.2, 5 mm Mg-ATP, and 100 units/ml PKA catalytic subunit). The membrane vesicles were stored at a protein concentration of 2–6 mg/ml at −75 °C until use.

### Preparation of CPX and DAX—

CPX was synthesized according to GMP (“Good Manufacturing Practice”) as part of our program for preparing CPX for clinical trials on cystic fibrosis patients. The CPX was solubilized at a concentration of 10 mm in dimethyl sulfoxide, and further diluted in dimethyl sulfoxide prior to dilution into the chamber solution. Prior to recording the effects of the drug, the contents of the chamber were mixed for 30 s using an internal mixing apparatus. The final concentration of dimethyl sulfoxide never exceeded 1%, and this amount of dimethyl sulfoxide alone was found to be entirely inactive, either on the naked bilayer or on incorporated CFTR channels. DAX was synthesized as described previously (23).

### Planar Lipid Bilayer Technology—

Planar bilayers were formed by applying a suspension of palmitoyloleoyl phosphatidylethanolamine and palmitoyloleoyl phosphatidylserine, 1:1, 50 mg/ml, each in *n*-decane, to a hole of about 100–120 *μ*m in diameter in a thin Teflon^™^ film separating two compartments that contained defined salt solutions (31). Channels were incorporated from a suspension of microsomes prepared from HEK293 cells expressing wild-type CFTR. The microsomes were added to the *cis* chamber in small aliquots, and incorporation occurred directly from the experimental solutions. Currents associated with CFTR channels were observed shortly after the bilayer system was exposed to protein kinase A (100 units/ml). The specific conditions include a KCl gradient (*cis* = 200 mm; *trans* = 50 mm), (1 mm) MgCl_2_, and ATP (2 mm) in *cis*, and 10 mm Tris/HEPES in *cis* and *trans*, adjusted to a final pH of 7.0. Single channel currents were recorded using a patch-clamp amplifier (AXOPATCH-1D equipped with a CV-4B 0.1–100 Bilayer headstage, Axon Instruments, Foster City, CA), and data were stored on magnetic tape using a pulse-code modulation/video cassette recorder digital system (Toshiba) with a frequency response in the range from direct current to 25,000 Hz.

### Statistical Evaluation and Channel Current Analysis—

Off-line analysis of the recorded CFTR channel activity was carried out using the software package pClamp 5.51 and 6. (Axon Instruments, Foster City, CA). Data base files were obtained from playbacks of the experimental records digitized using a 12-bit analog to digital converter (TL-1 DMA interface, Axon Instruments) using the fetchex subroutine. The channel current signal from the PCM-VCR was fed through a low pass filter (eight-pole Bessel 902 LPF; Frequency Devices Inc., Haverhill, MA) in series with the ADC module. The filtering level was set between 50 and 100 Hz. As a rule the data base used for open time and close time distribution corresponded to filtered recordings with a signal to noise ratio of ≥4:1.

## RESULTS

### Single Channel Recordings of CFTR—

HEK293 cells expressing a permanently transfected CFTR gene were grown and microsomes were prepared by differential centrifugation (30). Microsomal membrane vesicles carrying the expressed protein were then incorporated into a planar lipid bilayer, and ionic currents measured in the presence of a chemical and potential gradient. Currents associated with CFTR channels were observed shortly after the bilayer system was exposed to protein kinase A (100 units/ml). The specific ionic conditions, as described under “Materials and Methods,” were maintained throughout all the experiments described in this work. As previously reported, these channels are insensitive to DIDS (50 *μ*m), blocked by diphenylamine-2-carboxylate (300 *μ*m), and are selective for chloride (26, 27, 30).

**Fig. 1.**
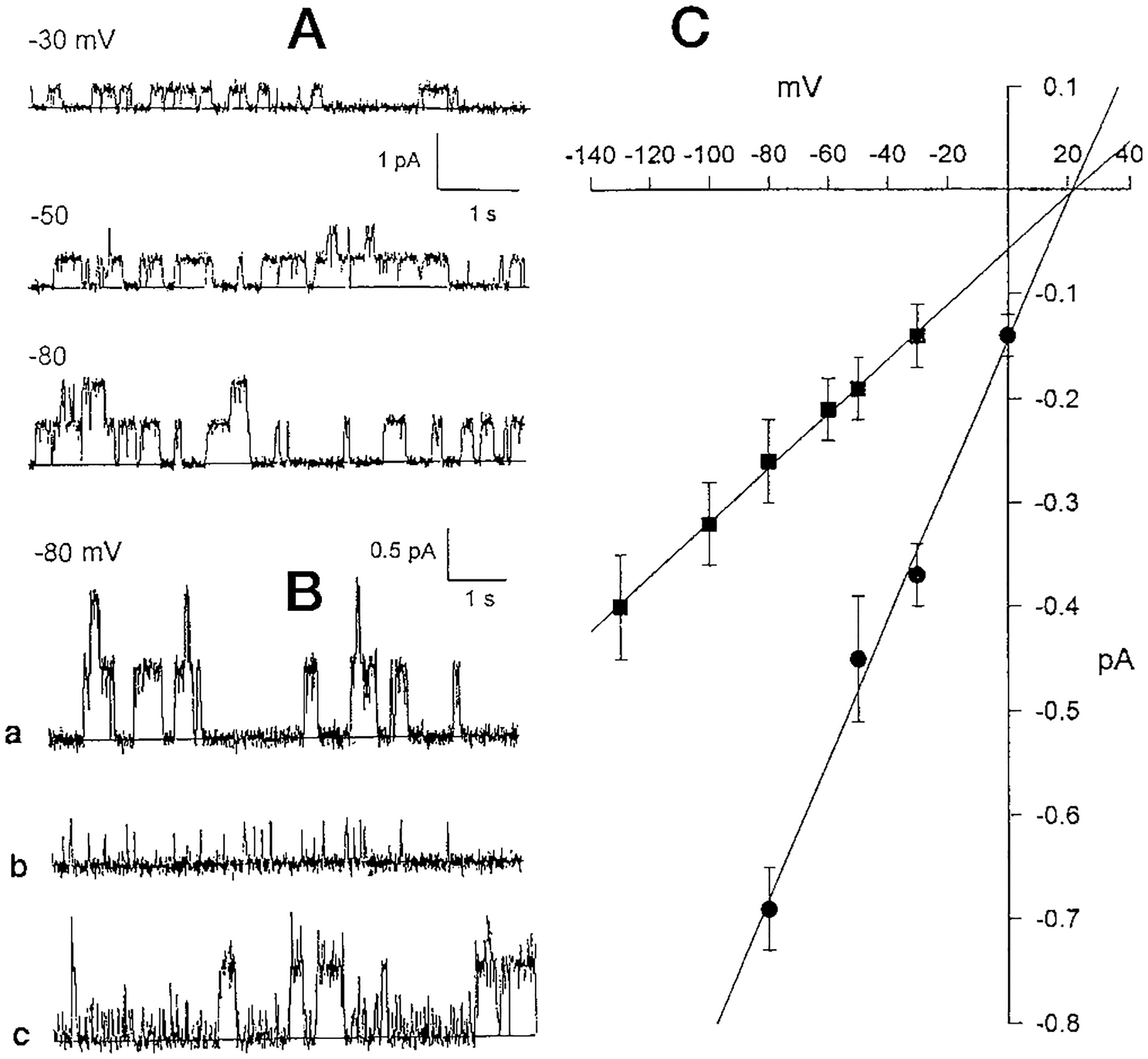
Characteristic ion channel activity from CFTR channels expressed in HEK293 cells. *A*, representative current records for CFTR. Three representative current records, taken at −30, −50, and −80 mV, demonstrate the most common types of current events observed after fusion of microsomal membrane vesicles from HEK293 cells to the lipid bilayer. At least two channels of about 7 pS conductance are observed in this example. The bilayer system is a KCl gradient (*cis*, 200 mm; *trans*, 50 mm), with ATP and PKA in the *cis* compartment. The voltage is given relative to the *cis* compartment. *B,* expanded time scale of segments of a CFTR current record taken at a membrane potential of −80 mV. *a*, the data demonstrate the simultaneous incorporation of two channels of identical conductance. The ionic conditions are the same as for *a. b*, the data demonstrate current events of smaller amplitude than those shown in *part a*. The ionic conditions are the same as for *A. c,* the data demonstrate interspersed current events from both the large and small CFTR channel. The ionic conditions are that same as for *A. C,* current-voltage relationship for large and small CFTR channels. Each point represents the arithmetic mean ± S.E. of at least 500 events measured at each potential. The slopes of the lines, calculated from regressions fit to the points, indicate conductances of 6.7 and 2.5 pS to the large and small currents events, respectively. The slope conductances are calculated as the regression fit of the points. Both regression lines intersect on the horizontal axis at the equilibrium potential of about 22 mV.

Fig. 1A, illustrates typical current events observed upon application of electrical potentials (−30, −50, or −80 mV) to the cis compartment. These events correspond to conductances of 7–10 pS, and additional current levels, double that of the first, are occasionally observed. These data indicate that more than one channel of the same type has been incorporated in the bilayer. In addition, we observe subconductances of about 2.5 pS, as described previously for CFTR channels (30). Fig. 1B shows the expression of several combinations of these large and small CFTR conductances. To better visualize the relationships a more expanded amplitude scale is employed for records taken at −80 mV. In Fig. 1B, *part a*) multiple larger current events are shown. In Fig. 1B, *part b,*, isolated smaller current events (smaller conductance) are shown, and in Fig. 1B, *part c*, mixtures of the larger and smaller current events are observed. The relationship between amplitude and voltage for this family of current events is shown in the appended I-V curve (Fig. 1C). The values of current in the I-V curves are calculated as the arithmetic mean ± S.E. The larger slope conductance (*solid circles*) is 6.7 pS, with an equilibrium potential of −21.5 mV. The smaller slope conductance (*solid squares*) is 2.6 pS, with an equilibrium potential of −22 mV. Thus on the basis of blocker sensitivity, chloride selectivity, and conductance states these channels correspond to CFTR activity.

### Influence of CPX on CFTR Channel Activity—

**Fig. 2.**
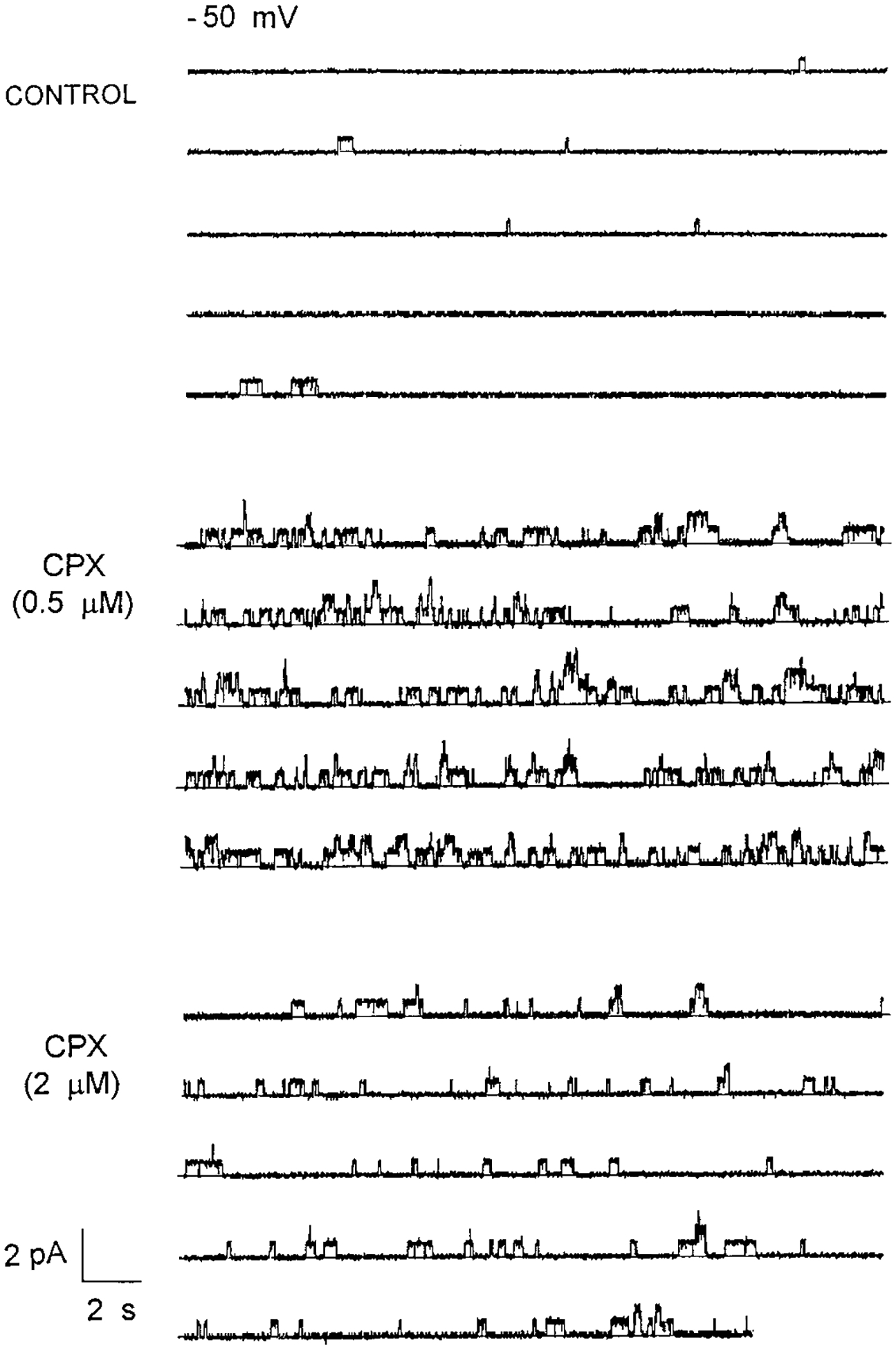
Effect of CPX on CFTR channel activity. *Upper panel,* control. Two minutes of continuous recording of a modestly activated CFTR channel, driven by a 50 mV membrane potential and a KCl gradient (200 mm *cis* and 50 mm *trans*). *Middle panel,* 2 min after the addition of CPX (500 nm) to the *cis* compartment. The channel activation is observed as an increase in the current activity both in number of opening events and multiple current levels. *Bottom panel,* further addition of CPX (up to 2000 nm CPX) to the *cis* compartment maintains the overall increased current activity.

CFTR was incorporated into the planar lipid bilayer, and the channel modestly activated by addition of PKA and ATP (see “Materials and Methods”), Fig. 2A. These data represent the control condition, in which the dominant motif is relatively low CFTR channel activity at the conductance level of 8.3 pS, and generated by a −50 mV driving force potential. Fig. 2B shows continuous recordings of CFTR channel activity, generated by the same driving force 5 min after 500 nm CPX was added to the *cis* side of the planar lipid bilayer system. The figure represents 2 min of continuous recording. The overall general activity is considerably increased, and the system exhibits multilevels of current that were not observed under control conditions. Upon elevation of the CPX concentration to 2 *μ*m (see Fig. 2C), the general pattern of activity is reduced relative to the maximum at 500 nm CPX. However, it remains higher than control. This type of “bell-shaped response” is precisely what would have been predicted from our previously published results with the effect of CPX on chloride efflux from CF cells (19, 23). A kinetic analysis of these data, shown below, indicates that the effect of CPX is to increase the number and duration of open events of CFTR channels without modifying their conductance and selectivity.

### I-V Curve of CFTR Channels in the Presence of CPX—

**Fig. 3.**
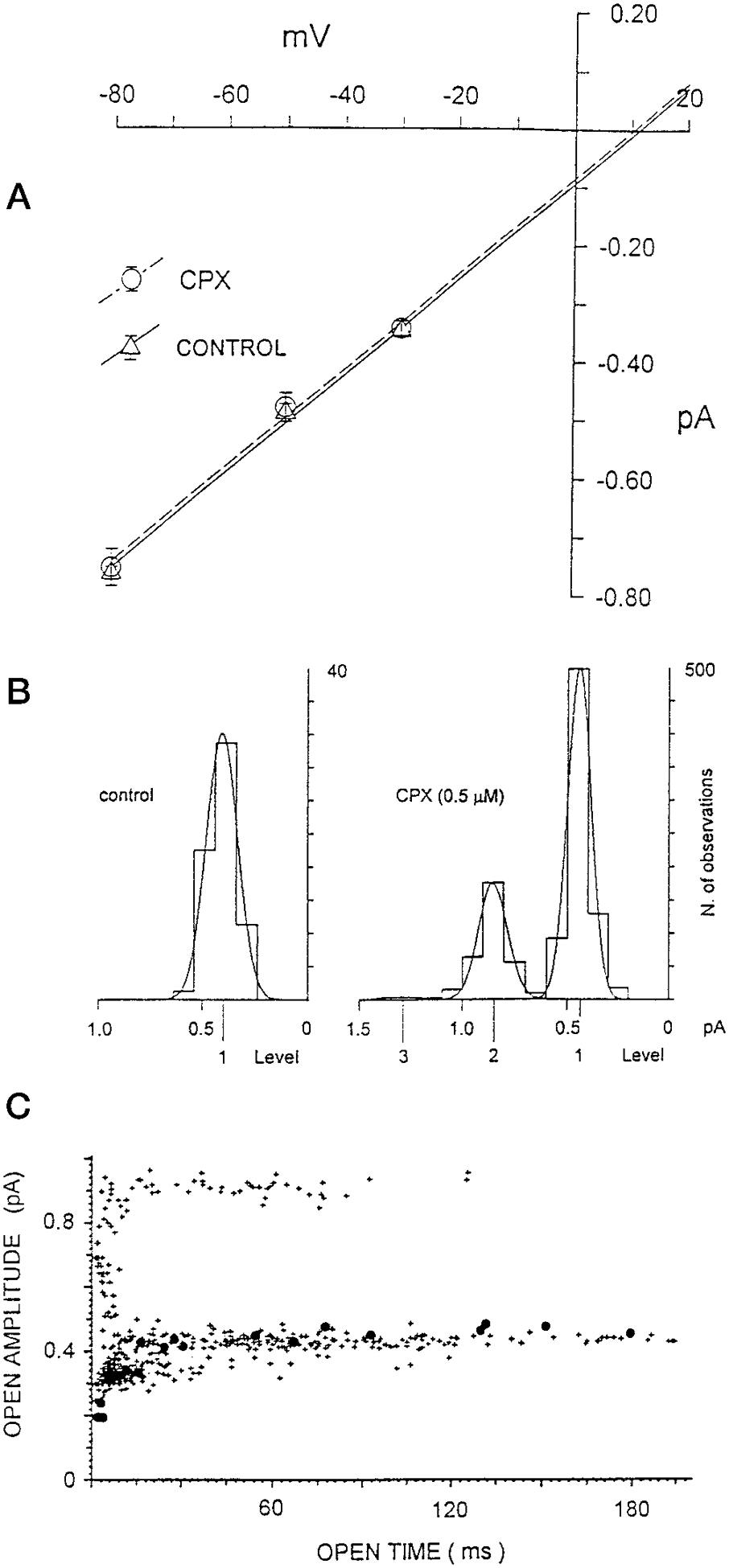
CPX effects on CFTR channels. *A*, influence of CPX on the I-V relationship of CFTR channels in a KCl gradient (200 mm *cis* and 50 mm *trans*). The relation between mean amplitude of unitary current events and the membrane voltage in control and CPX treated conditions is fitted by linear regression. The slope conductances are both 8.2 pS, and the intersections on the *horizontal axis* indicate current equilibrium potentials around 12 mV. *Symbols* are as indicated. *B,* current events amplitude histograms from records at −50 mV membrane potential. *Left panel*, amplitude distribution of unitary current events during control conditions. *Right panel*, amplitude distribution of the all recorded current events in the presence of 500 nm CPX in the *cis* compartment. Gaussian fit indicates that mean amplitude of the unitary events is 0.48 pA for −50 mV holding potential, in control and in the presence of the drug. *C,* scatter plot of the amplitude of the events in both control (*filled circles*) and CPX (*cross symbols*) as a function of duration events. Currents events of any open time duration, both in control and in the presence of the drug, are positioned around a mean current amplitude (or multiple) of 0.48 pA. Except for current events of very short duration (less than 20 ms) whose amplitudes are affected by the filtering conditions, no intermediate current amplitude events are observed.

The I-V relationship for CFTR channels under control and 500 nm CPX conditions are shown in Fig. 3A. To prepare these data we plotted the arithmetic mean current amplitude of unitary events recorded at different electrical potentials (≥59 events in control conditions and ≥257 events in the presence of CPX, for each potential). Regression lines fit for the two conditions are statistically identical, indicating that the slope conductances (8.2 pS) and equilibrium potentials (12 mV) for ion fluxes are the same for both conditions. This indicates that the ionic selectivity and the unitary conductance of CFTR channels are not affected by the interaction with the drug. Rather, the increased current activity of the system must be due to a direct effect of CPX on the kinetics of the CFTR channel behavior.

### Influence of CPX on Amplitude of CFTR Current Levels—

The action of CPX on the CFTR conductance can be most clearly visualized by construction of amplitude histograms from current records. Fig. 3B, *left panel*, shows such an amplitude histogram from a control record summarizing the distribution of amplitudes at −50 mV. The Gaussian distribution of the mean amplitudes of these events gives an average amplitude of 0.48 pA (59 unitary events). In the presence of 500 nm CPX (Fig. 3B, *right panel*) the amplitude histogram of all events occurring during 2 min recording reveals that the events are distributed in three levels. The principal Gaussian peak (number 1)(749 events) is very similar to that of the control in the absence of CPX. The additional peaks (number 2 (257 events) and number 3 (9 events)) are exact multiples of the principal peak. These data indicate that the CPX-induced increase in CFTR activity are likely to be additional CFTR channels.

A second approach to the analysis of CPX action on the CFTR channel is to create a scatter plot of amplitudes as a function of duration of the events. The *large solid dot symbols* in Fig. 3C are the values of current amplitudes at −50 mV under control conditions. The *cross symbols* are the values of current amplitude after the application of 500 nm CPX. These data indicate that whatever the duration of the event the mean amplitudes are the same, regardless of the presence or absence of CPX. Furthermore, the control condition and the lower level measured in the presence of CPX appear to be quite coincident. However, in the presence of CPX, a very well defined second level occurs at an amplitude which is twice the control. Furthermore, this level is constant regardless of the duration of the event. The conclusions from both analytic approaches similarly indicate that CPX increases the number of active CFTR channels. Data shown later (see Fig. 7) indicate that CPX also increases the open time probability.

### Effect of CPX on the Activity of the 2.5 pS CFTR Conductance—

**Fig. 4.**
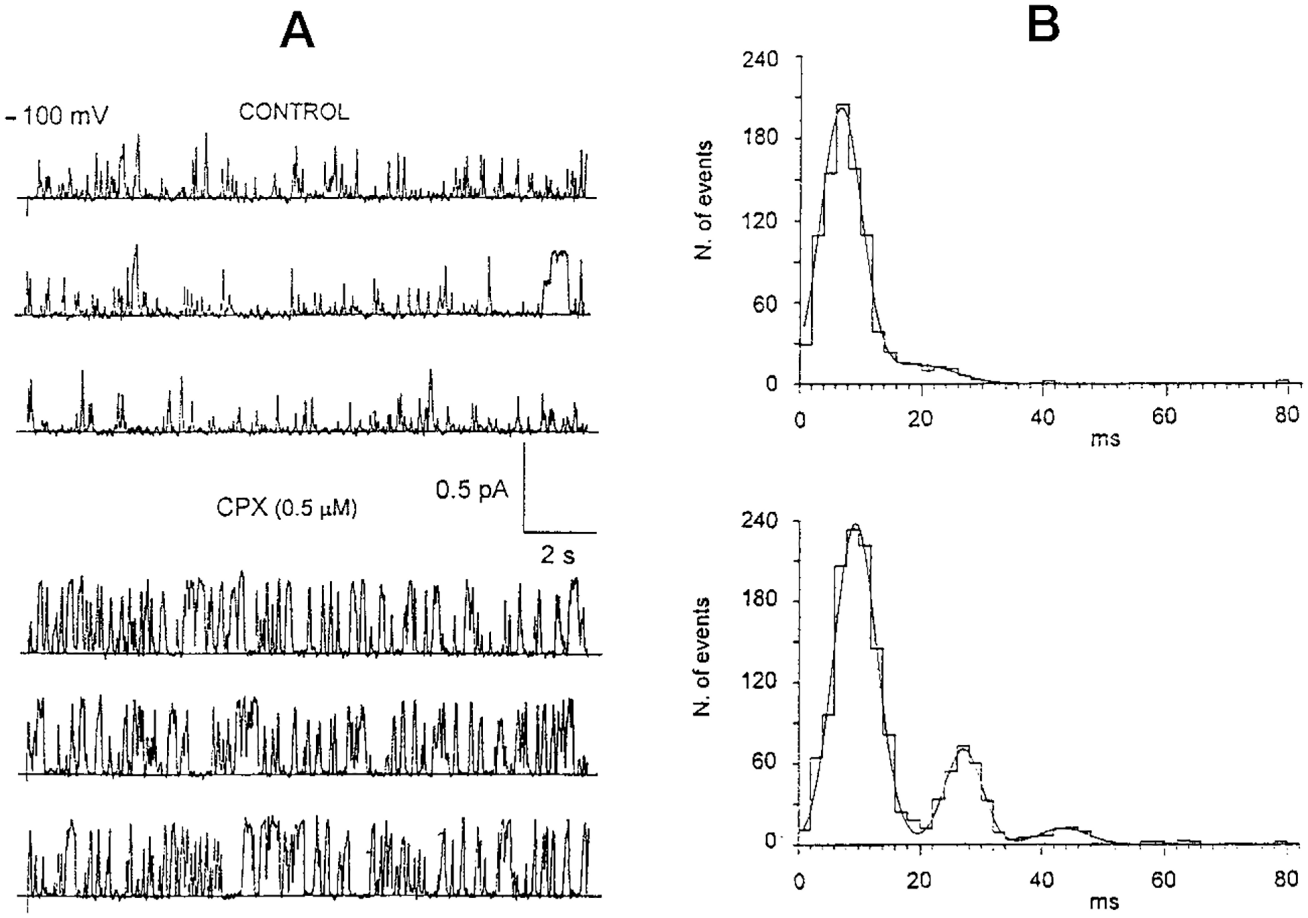
Influence of CPX on small CFTR conductance. *A*, effect of CPX on the activity of the 2.5 pS conductance associated with the expression of CFTR. *Upper panel*, control channel current events generated by −100 mV potential. *Lower panel*, same channels as in the *upper panel*, after addition of CPX (500 nm). *B,* influence of CPX on open time distributions of current events from the 2.5 pS ionic conductance associated with expression of CFTR. *Upper panel*, in control conditions the current events are distributed into two population. Two Gaussian fit to the data indicates peak durations of 8 and 24 ms. *Lower panel*, distribution of channel current events in the presence of 500 nm CPX, fitted to three Gaussian distributions. The peak durations are 9, 27, and 44 ms.

As mentioned above, the smaller 2.5 pS conductance associated with CFTR expression can, on occasion, be incorporated as a single channel in isolation from the larger 7–10 pS CFTR conductance. We therefore took advantage of several such instances to study the influence of CPX on the smaller conductance. Addition of CPX also increased the overall activity of the 2.5 pS conductance. For a better signal to noise ratio, we analyzed this increased activity at a large potential. As shown in Fig. 4A, control conditions at a membrane potential of −100 mV (*upper panel*) are characterized predominantly by brief spikes, which under the 50 Hz filtering conditions give an apparent average open time of 8 ms and an apparent arithmetic mean amplitude of 0.08 pA. After the addition of 500 nm CPX the activity of the channel increases profoundly, with the apparent arithmetic mean amplitude increasing to 0.14 pA (Fig. 4A, *lower panel*). The effect of CPX is to increase the duration of single events. Quantitatively, the open time probability increases from about 8% in control conditions to 35–38% in the presence of CPX. Since the control amplitude of 0.08 pA is the average spike amplitude observed under high filtering conditions (50 Hz), the apparent increase in current amplitude mediated by CPX may not be the real effect of the drug. This is because the amplitudes of longer events are less attenuated by the filtering condition. Thus, alternatively, the apparent increase in current amplitude could also be a consequence of the ability of CPX to prolong the open time of individual events.

### Influence of CPX on Open Time Distributions of the 2.5 pS CFTR Conductance—

To test the latter hypothesis we plotted open time histograms of the 2.5 pS CFTR conductance for both control and 500 nm CPX conditions. Channel activity of the 2.5 pS conductance under control conditions is characterized by predominantly brief spikes with an average duration of 8 ms (see *upper panel* of Fig. 4B). A minor peak is observed at approximately 24 ms. In the presence of CPX (see *lower panel*, Fig. 4B) the increase in open time duration is not monotonic but is distributed into three main Gaussian populations, with peak durations of 9, 27, and 44 ms, respectively. However, we observe that the 27 ms population in the CPX treated condition is actually present to a very minor extent in the drug-free condition (*viz*. the 24 ms population in the control histogram in the *upper panel* of Fig. 4B). These data therefore suggest that the CPX may activate or modify existing 2.5 pS CFTR channels, rather than merely recruit cryptic or new CFTR channels. We conclude that these data support the concept that addition of CPX not only increases the number of events, but also their duration.

### Influence of DAX on CFTR Channel Activity—

**Fig. 5.**
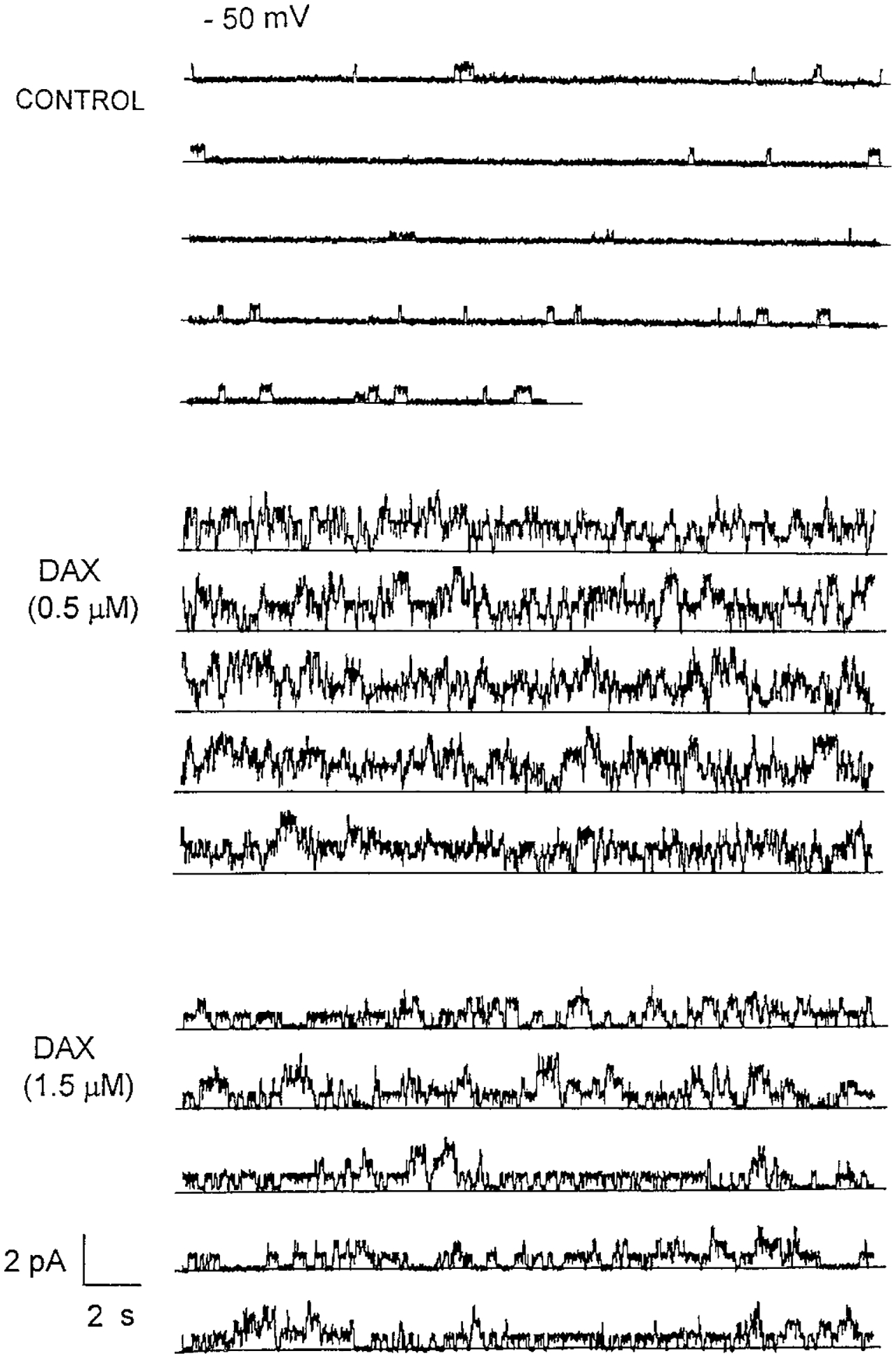
Influence of DAX on CFTR channels. *Upper panel,* control. Two minutes of continuous recording of a modestly activated CFTR channel, driven by a 50 mV membrane potential and a KCl gradient (200 mm *cis* and 50 mm *trans*). *Middle panel*, after the addition of DAX (500 nm) to the *cis* compartment, a potent activation is observed as an increase in the current activity both in number of opening events and multiple current levels. *Lower panel*, further addition of DAX (up to 2000 nm) to the *cis* compartment maintains the overall increased current activity.

DAX is also a potent activator of chloride conductance in CF cells, much like CPX, which we hypothesized might also activate CFTR channels. To study this possibility we followed the same protocol used to study CPX. Therefore, prior to the addition of DAX we established an active CFTR channel using PKA/ATP (see “Materials and Methods”). We then added DAX at various concentrations and studied the channel activity at various membrane potentials. In all conditions, addition of DAX produced a profound increase in basal channel activity. Fig. 5A summarizes one of those results for a driving force potential of −50 mV. The data representing the control condition show a dominant motif of relatively low activity at the level of 7–10 pS. The 2.5 pS conductance is absent from this example. DAX (500 nm) is then added to the *cis* side of the planar lipid bilayer system. As shown in Fig. 5B, the number of channel events is considerably increased. By inspection, at least four different levels can be observed. Furthermore, the apparent duration of channel events appears elevated. Then, when the DAX concentration is increased to 1.5 *μ*m (Fig. 5C), the general pattern of activity is only slightly reduced compared with the more profound suppressive effect of a similar concentration of CPX (compare with Fig. 2C).

### Comparison of CPX and DAX Effects on CFTR Channel Activity—

**Fig. 6.**
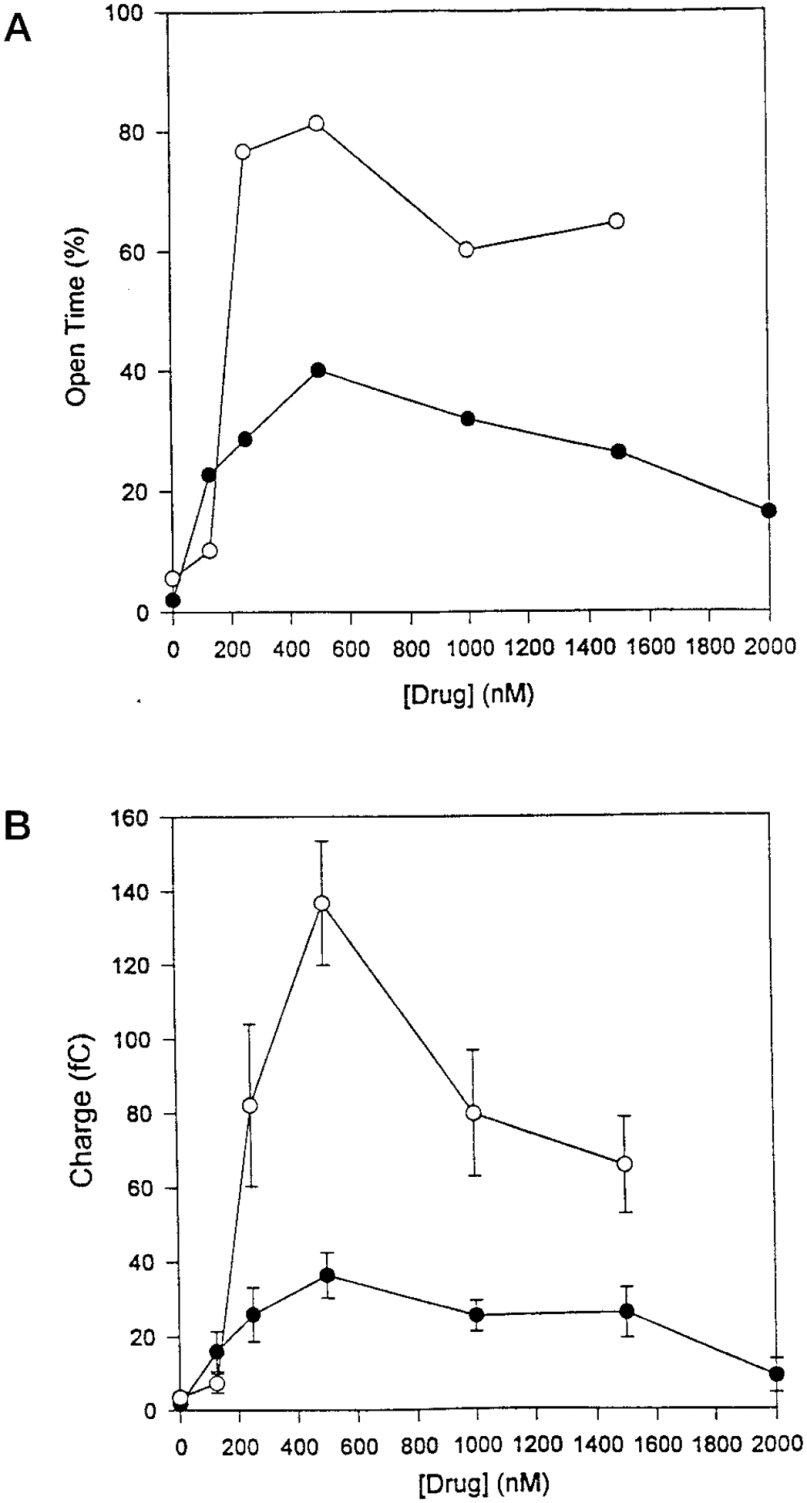
Concentration dependence for CPX and DAX on CFTR channel activation. *A*, influence of CPX and DAX on open time of CFTR channels. Drugs were added to the *cis* compartment in a stepwise manner. Data were collected 3 min after drug addition. Open time probability was estimated from 2 min of subsequent data. *B*, influence of CPX and DAX on negative charge conducted by CFTR. Experiment was conducted exactly as in *part A*. Average charge conducted was calculated by integrating serial 15-s intervals over a 2-min period of current recording.

The data above suggest that CPX and DAX are similar in their ability to activate the CFTR channel, but that they differ in terms of potency and kinetics. To further examine this possibility we titrated CPX and DAX in a stepwise, dose-escalating fashion on CFTR channels in the large 7–10 pS range. Fig. 6 shows drug titrations on two different channel incorporations in which it was possible to follow the activity of each of the channels systematically, up to 2000 nm for CPX or 1500 nm for DAX. Although the drugs were tested on different channels, the control open probabilities for the two channels were very similar before the addition of the drug (2 and 5% for CPX and DAX, respectively). Therefore, in Fig. 6 we superimposed all the resulting data in the same plot in an attempt to compare the effects of the two drugs.

We approached the analysis of the drug effects in two ways. In Fig. 6A, we plot the first level open time probability as a function of drug concentration. This indicates the fraction of time that any one of the activated channels is conducting, regardless of the total number of channels simultaneously open. While this solution does not give the open time probability of each individual single channel, the importance of this approach is that it serves to emphasize quantitatively the increased activity by CPX and the relatively more efficacious DAX. CPX causes an apparent open time maximum for CFTR at about 500 nm. At higher concentrations of CPX (500–2000 nm) the open time probability begins to decline monotonically. By 2000 nm CPX, the open time probability has declined to about 50% of the maximum activation obtained with 500 nm CPX. Using the same analytical approach for DAX, we find that the CFTR channel activation is clearly more efficacious than that by CPX (see Fig. 6A). By the 250 nm DAX concentration point, maximum activation had already been achieved. At higher concentrations of DAX (500–1500 nm) the open time probability remains within 25% of the maximum activation obtained with 250 nm DAX.

However, this approach does indeed underestimate the contribution of the additional channels simultaneously activated by the drugs. An alternative approach is to analyze drug-induced changes using integration of the total current. We therefore averaged the amount of negative charge conducted across the membrane in consecutive 15-s interval after achieving a steady state in the level of activation. Steady state was achieved in approximately 3 min, after the new concentration levels were established. As shown in Fig. 6B, the CPX and DAX concentrations needed to cause a maximum in charge conducted is similar, about 500 nm. DAX efficacy appears to decline from a maximum at 250 nm (Fig. 6A) to 500 nm (Fig. 6B) because the channels active in the DAX induction are underappreciated by the approach followed in Fig. 6A. However, DAX is still profoundly more efficacious than CPX in terms of inducing chloride ions conduction across the membrane.

### Influence of PKA and CPX on CFTR Channel Kinetics—

**Fig. 7.**
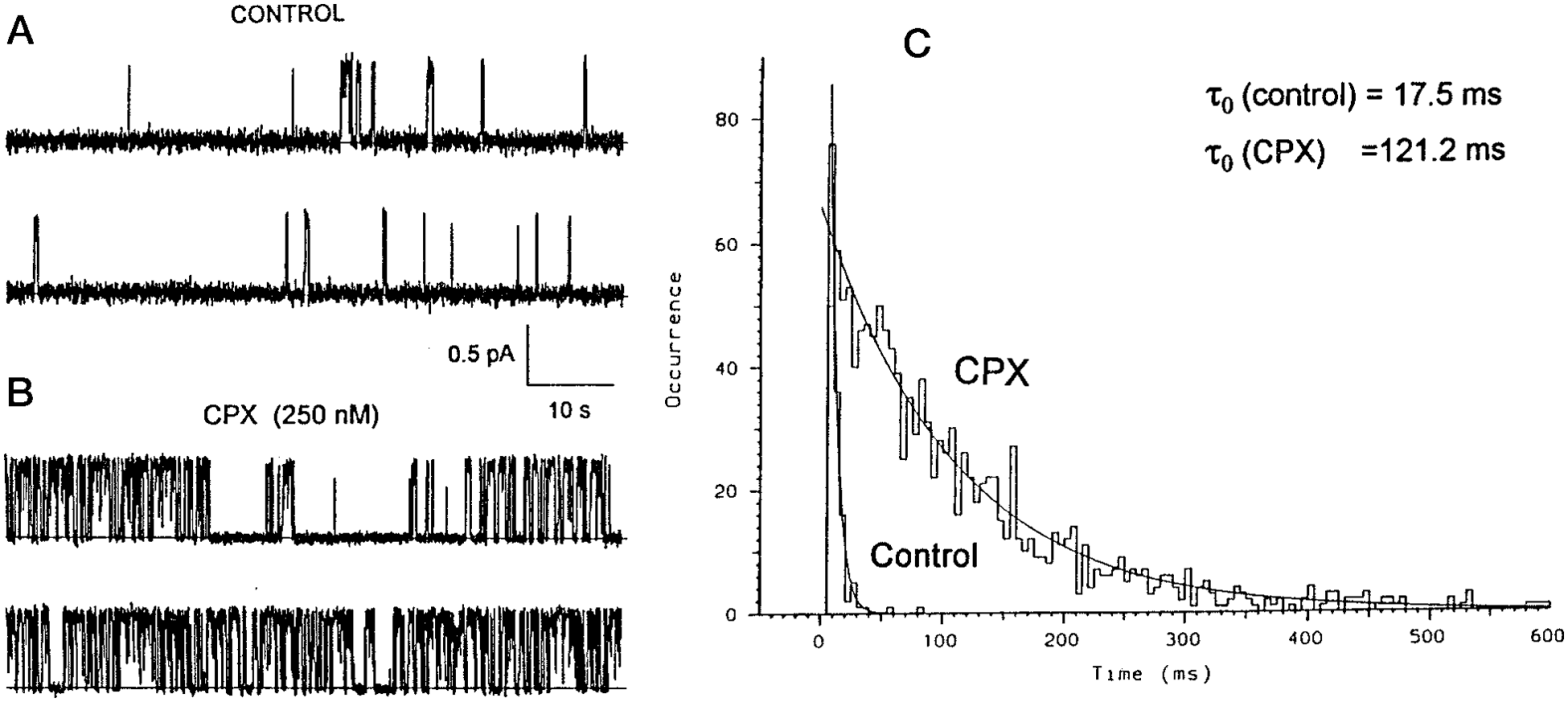
Effect of CPX on mean open time of CFTR channels. *A*, continuous recording of CFTR channel activity after removal of PKA and ATP from the chamber. *B,* CFTR channel activity from *part A* after equilibrium activation by CPX (250 nm). Conditions include a KCl gradient as described under “Materials and Methods,” and a −80 mV electrical potential in *cis. C*, histograms of mean open time for CFTR channel under the conditions for *A* and *B. Fitted lines* give values of *τ*_*o*_. The control value is 17.4 ms; the CPX value is 121 ms.

To further evaluate the possibility of direct activity of CPX on the CFTR channels, we activated the channels in the usual way, and then removed PKA and ATP from the chamber. As shown in Fig. 7A (see control currents), basal activity was sustained. Indeed, we saw no substantive change in the open probability of CFTR channels. This observation is not surprising since it is widely appreciated that once the channel is phosphorylated removal of PKA has no effect on sustained activity (38). Dephosphorylation is a counteracting process which is known to reduce CFTR activity (38, 39). Thus the sustained activation of CFTR after removal of PKA in our system indicates that the functional phosphatase activity incorporated with these channels is minimal.

Upon addition of CPX (250 mm) to the control channels shown in Fig. 7A, a substantial increase in activity is observed (Fig. 7B). By inspection it is apparent that one of the effects of CPX is to increase the burst duration. In the experiment shown, there was an initial period of time in which only a single channel was recorded. We therefore took advantage of this opportunity to measure the effect of CPX on single channel kinetics of these large CFTR channels. Histograms of these opening events, before and after CPX addition, is shown in Fig. 7C. It is very clear that CPX causes a remarkable increase in the average duration of the openings. Values of *τ*_*o*_ are 17.5 ms for control, and 121 ms for CPX.

## DISCUSSION

The experimental results presented here provide evidence that CFTR chloride channels, incorporated in planar lipid bilayers and activated by pKA and ATP, can be further activated by the addition of the xanthines CPX or DAX. This enhanced activation appears to be the effect of direct interaction of these drugs with the CFTR channel molecules. Earlier data leading to these studies with intact cells had shown that CPX could activate chloride efflux (17–19) or chloride current (20) from cells expressing either wild-type or mutant (ΔF508) CFTR, respectively. In addition, these drugs have been shown to bind selectively to a specific domain (NBF-1) of the CFTR molecule (24, 25). However, until the present study a direct connection between CPX or DAX binding and CFTR channel activation, *per se*, has been missing. The data presented in this work therefore provide strong evidence that the mechanism of activation is indeed very likely to be by direct interaction between CPX or DAX and the CFTR molecule.

CFTR channel activation, measured as a pronounced increase in the total open time probability or total conductive time, is a combined result of an increase in the frequency and in the duration of channel opening events. The observed increased frequency in the number of events could have several origins, since the source of CFTR channels are microsomes from transfected cell membranes which fuse to the lipid bilayer. We envision at least three possible activation mechanisms to explain the observed overall channel activation. First, the drugs may further activate an already activated channel. For example, we do observe an increase in the frequency and duration of unitary CFTR channel events after drug addition. Second, the drugs might activate otherwise inactive but incorporated channels. The evidence is that multiple channels become evident only after the drugs are added. However, all channels in the experimental chamber are potentially activated since they are permanently bathed in PKA and ATP, and we do not see activation by the drugs if channels have not been previously activated by PKA. Finally, it is possible that the drugs might facilitate the fusion to the bilayer of new microsomes carrying active channels. For example, CFTR can facilitate endosome-endosome fusion (32), and recombinant NBF-1 promotes phosphatidylserine liposome interaction (25, 33). However, we do not detect new conductances after drug addition, which might have supported this third possibility. It therefore follows that the available data do not rule out any of these mechanisms. However, the data presented here do tend to favor the possibility that CPX and DAX further activate otherwise active CFTR channels by direct interaction with the protein.

From a different perspective, recently published radioligand binding data have indicated that both CPX and DAX bind with high affinity to the recombinant first nucleotide-binding fold domain (NBF-1) of CFTR (24). In addition, the ΔF508-NBF-1 binds both ligands with substantially greater affinity than wild-type NBF-1 (24). Thus, taken together both the binding data and the channel data presented here provide strong arguments for the ability of CPX and DAX to activate CFTR by direct interaction with the CFTR protein. These results also further exclude the possibility that simultaneous actions of CPX on adenosine A_1_ or adenosine A_2_ receptors might be responsible for the unusual kinetic activity on CFTR function. An important pharmacologic difference between CPX and DAX is that while CPX is a quite potent adenosine A_1_ antagonist, DAX is relatively poor. Yet they both activate CFTR in cells and bilayers with intrinsic but proportional differences in both systems.

Finally, we can speculate about the possible mechanisms by which these xanthine drugs might activate CFTR channel activity. The data in Fig. 7 indicate that to observe the channel, continued presence of PKA in the chamber is not needed once the channel is phosphorylated. Furthermore, the continued presence of PKA is not needed to observe channel activation by CPX. The sustained activity after removal of PKA indicates that endogenous phosphatase activity is minimal, and that the CPX activation is probably not through inhibition of this otherwise minimal activity. Thus CPX activation does not appear to be mediated by action on ancillary activation or inhibition systems, leaving only direct action on the CFTR protein as the likely mechanism. In searching for the most likely site of CPX interaction on CFTR, attention could immediately turn to the NBF-1 domain of CFTR for a variety of circumstantial but relevant reasons. First, both CPX and DAX also bind with high affinity to the recombinant first nucleotide-binding fold domain (NBF-1) of CFTR, in the vicinity of F508 (24). Second, the NBF-1 domain is immediately contiguous with the cytosolic aspect of the sixth transmembrane domain of CFTR (TMD6), and Akabas and colleagues (34, 35) have shown that this domain is important in defining the selectivity filter of CFTR. This sequential contiguity provides a structural basis for understanding how ATP might affect channel function, and might also provide a basis for direct CPX or DAX actions on the channel. Finally, the fact that NBF-1 itself interacts intimately with artificial (31) and natural membranes (36, 37) could provide the energetic basis for hypothetical close interactions between TMD6 and NBF-1 within or near the plasma membrane.

## Footnotes

1 The abbreviations used are: CF, cystic fibrosis; CFTR, cystic fibrosis transmembrane conductance regulator; CPX, 8-cyclopentyl-1,3-dipropylxanthine; DAX, 1,3-diallyl-8-cyclohexylxanthine; PKA, protein kinase A; DIDS, 4,4′-diisothiocyanostilbene-2,2′-disulfonic acid; NBF, nucleotide-binding fold.
